# A simple experiment to improve adherence for taking the oral contraceptive pill: An exploratory study of behavioural mechanisms

**DOI:** 10.1111/bjhp.12788

**Published:** 2025-03-03

**Authors:** Caitlin Liddelow, Barbara A. Mullan, Mark Boyes

**Affiliations:** ^1^ School of Psychology, Faculty of Arts, Social Sciences and Humanities University of Wollongong Wollongong New South Wales Australia; ^2^ EnAble Institute, Faculty of Health Sciences Curtin University Perth Western Australia Australia; ^3^ School of Human Sciences University of Western Australia Perth Western Australia Australia; ^4^ School of Population Health, Faculty of Health Sciences Curtin University Perth Western Australia Australia

**Keywords:** behaviour change, education, habit, menstrual cycle, pregnancy

## Abstract

**Objectives:**

Full adherence is imperative to ensure the prevention of unintended pregnancies, which have serious health and financial impacts on women. Previous research has identified the importance of cues (habit‐based) and providing information from a credible source (non‐habit‐based) in facilitating adherence.

**Design:**

A 6 week experiment was developed to increase adherence to the pill (primary outcome) as well as increase habit strength for taking the pill (secondary outcome).

**Methods:**

A sample of Australians who menstruate (*N* = 77, *M*_age_ = 25.18, *SD* = 3.49) were randomly assigned to one of four conditions: (1) a control group, (2) receiving information from a credible source, (3) implementing a daily cue, and (4) receiving both the information and instructions to implement a cue. At baseline and six‐weeks, participants completed two measures of adherence to the pill, and a measure of habit strength.

**Results:**

Mixed‐model ANOVAs revealed no significant changes in adherence to the pill across conditions, over time. There was a significant increase in habit strength over time (*η*2 = .11), across all conditions (*η*2 = .11). However, these changes did not significantly differ by condition (*p* = .071).

**Conclusions:**

These findings suggest participating in an experiment, regardless of condition, may make taking the pill more salient and thus increase habit strength. It also suggests that providing information from a credible source nor associating taking the pill with a daily cue substantially increased adherence. However, adherence was positively skewed and therefore these findings need to be further explored with individuals with lower adherence.

**Statement of Contribution**

**What is already known on this subject?**

- Full adherence to the pill is rare, with 50% of users missing a dose of their pill at least once a month.
- Information from a credible source, and habit have both been shown to improve adherence to medications.

**What does this study add?**

- Providing information from a credible source increased habit strength for taking the pill more than cue associations.
- Trying to change behaviour for a non‐novel behaviour is difficult.

## INTRODUCTION

Unintended pregnancies are a serious public health issue, with a 2022 report finding that unintended pregnancies are associated with financial and social impacts on women, employers, out‐of‐home carers and the Government (Organon & HTAnalysts, 2022). They are also said to directly cost an estimated AUD $2.2 billion, with 91% of this being paid for by the Government and the remaining 9% paid by the women experiencing the unintended pregnancy (Organon & HTAnalysts, 2022). In addition to the financial impacts, unintended pregnancies can negatively impact the woman, child and their families. For instance, a review of the literature found that unintended pregnancy was associated with a higher likelihood of experiencing adverse birth outcomes such as congenital anomalies, premature delivery and low birth weight (Gipson et al., 2008). Non‐adherence to the oral contraceptive pill is a large contributor to experiencing an unintended pregnancy, with some studies reporting that approximately 73% of women were actively taking the oral contraceptive pill when they experienced an unintended pregnancy (Coombe et al., 2016).

According to the United Nations (2019), the oral contraceptive pill (otherwise known as “the pill”) is one of the most widely used methods of contraception globally, with an estimated 151 million users. In Australia, approximately one‐third of women between 16 and 49 years currently take the pill as their main form of contraception (Richters et al., 2016). In addition, it is expected that nearly 80% of women will use the pill for contraception at some stage in their life (Richters et al., 2003). Full adherence^1^ to the pill results in it being 99.7% effective at preventing pregnancy, however, with typical adherence (e.g., missing doses or not taking at the same time every day), the effectiveness of the pill is reduced to 91% (Trussell, 2011). Studies have shown that full adherence (taking at the same time, every day) is rare, with approximately 50% of users missing or forgetting to take a dose of their pill at least once a month (Molloy et al., 2012; Rosenberg et al., 1998). Recent research suggests that one‐third of women unintentionally forgot to take their pill in the previous week (Fumero et al., 2021), which could lead to experiencing an unintended pregnancy. One way to possibly improve unintentional non‐adherence (Phillips et al., 2013) to the pill, and reduce the number of unintended pregnancies, may be through making the behaviour habitual.

Previous research in adherence has shown a significant positive association between habit strength and medication adherence (Badawy et al., 2020; Hoo et al., 2019; Liddelow et al., 2022). In other words, people who report a higher habit strength for taking the pill were also more likely to report better adherence (Murphy et al., 2018). More specifically, habit strength has been shown to be a strong predictor of unintentional non‐adherence (Phillips et al., 2013). There is an ongoing debate in the literature around what exactly defines a ‘habit’, with some experts believing that a habit is the outcome of a learnt cue‐behaviour association, how fast a behaviour becomes automatic or the cue‐behaviour association itself (Gardner, 2015; Gardner et al., 2024; Wood & Rünger, 2016). In line with social cognition theories (e.g., Temporal Self‐Regulation Theory; Hall & Fong, 2007), and previous research that suggests that habit is a determinant of behaviour rather than a type of behaviour, (e.g., Gardner, 2015; Gardner & Lally, 2013; Phillips & Mullan, 2023), we define habit as “a cognitive process whereby a contextual cue automatically triggers an impulse to act, based on a cue‐behaviour association learnt through consistent repetition in the presence of the cue” (Gardner et al., 2024, p.3). This consistent repetition is then said to lead to a habitual behaviour, which can then sometimes lead to the behaviour being maintained in the long‐term (Kwasnicka et al., 2016).

The importance of cues in predicting and assisting engagement in healthy and unhealthy behaviours has been shown in previous research, such as in everyday nutrition behaviours (Keller et al., 2021), binge drinking episodes (Murray & Mullan, 2019), teeth flossing (Judah et al., 2013), and sugar‐sweetened beverage consumption (McAlpine & Mullan, 2022). Cues were initially broadly classified into two domains: internal (e.g., wheezing) and external cues (e.g., media advertisements), however, more recently it has been proposed there are five domains: physical (e.g., media advertisement, alarm), sensory (e.g., certain smell), social (e.g., spending time with certain friends), internal (e.g., sleepiness), and emotional (e.g., feeling sad; Booker & Mullan, 2013; Mullan & Novoradovskaya, 2018).

More specifically, cues are often identified as being important in improving medication adherence, as reported in both qualitative studies (Kelly et al., 2014; Liddelow, Mullan, & Boyes, 2020; Liddelow, Mullan, Boyes, & McBride, 2020), and quantitative studies (Liddelow et al., 2021; Stawarz et al., 2016). The importance of cues in adherence to specific medications have also been experimentally explored, with promising results (Phillips et al., 2021; Rigsby et al., 2000; Rosen et al., 2004). However, further research into the role of cues using experimental research is warranted, to fully understand its ability to facilitate adherence behaviours to a range of different medications.

As full adherence to the pill is imperative to ensure its success in preventing pregnancy, exploring the possibility of pairing the pill with a daily cue may be a promising avenue to explore in attempts to make taking the pill a habitual behaviour, and in turn, improve adherence. However, research suggests that behaviours (such as taking medication) may be more difficult to become habitual if the reward for doing the behaviour is distal (Mullan & Novoradovskaya, 2018; Phillips & Mullan, 2023a). In the case of the pill, users may not experience the benefit of the pill unless they are engaging in sexual practices where the risk of becoming pregnant is high (e.g., not using physical protection such as a condom). Thus, taking the pill may be more difficult to become a habitual behaviour because the ‘benefit’ is not experienced immediately. Similarly, there has also been research that suggests that habit alone may not be sufficient for long‐term maintenance of behaviour change (Gardner et al., 2024; Lally & Gardner, 2013), despite it being an important predictor of adherence (Phillips et al., 2013), and that other cognitive or more intentional processes may be important (Gardner, 2015; Kwasnicka et al., 2016). Due to this, it is important to explore (and compare) processes by which adherence to the pill can be improved, and potentially maintained, that do not involve making the behavioural habitual.

One relatively easy mechanism to employ that is said to improve medication adherence is through educating individuals using credible sources and information. Having a greater level of understanding of medications and their usage is linked to greater adherence as it is said to motivate people to adhere (Al‐Qazaz et al., 2011; Horne & Weinman, 1999; Mekonnen & Gelayee, 2020). Research exploring this association with adherence to the pill has also shown that greater understanding of the pill, its uses, mechanisms of action and side effects are positively associated with better adherence and continuation of the pill (Hall et al., 2014; Liddelow, Mullan, & Boyes, 2020). This supports research by Kwasnicka et al. (2016) who identified that social influences, such as having the correct skills and knowledge to effectively engage in a behaviour, can positively influence maintenance of the behaviour.

Furthermore, information provided about the oral contraceptive pill, whether in the form of a leaflet from a pharmaceutical company or spoken directly by a trained medical professional, often makes it explicit that for the pill to be the most effective, it must be taken at the same time every day (American College of Obstetricians and Gynecologists, 2019). This instruction may lead to engagement in planning strategies to assist them in adhering (Bandura, 1997). This idea is in line with Lally and Gardner (2013) who outline the four stages of habit formation (i.e., intention formation, intention to action, repetition and cue‐response association). They suggest that an individual needs to translate their intention into action through the use of self‐regulatory tools, such as becoming more knowledgeable about a behaviour to sufficiently plan for it, before they can begin forming the habitual behaviour. In contrast, there is also research that suggests that education/knowledge alone is insufficient to lead to positive behaviour change (Arlinghaus & Johnston, 2018), and that it needs to be paired with other behaviour change mechanisms. However, this has yet to be explicitly explored. Therefore, providing information from credible sources, such as a pharmaceutical company, as a mechanism for improving adherence and habit strength for the pill, lends itself to being further explored both as an individual mechanism and in combination with other mechanisms (i.e., cues).

### The present study

The aim of this study was to experimentally explore two different mechanisms that were hypothesised to increase adherence to the pill, and increase habit strength, namely implementing a cue to pair with the behaviour, and providing information from a credible source. This experiment is explicitly targeting the implementation component of adherence, which is “the extent to which a patient's actual dosing corresponds to the prescribed dosing regimen, from initiation (the first dose) to discontinuation (the last dose)” (Vrijens et al., 2012, p. 696). Using a 4 (experimental condition) x 2 (time points, baseline and follow‐up) repeated measures design we recruited people who menstruate, and who self‐identified as not very good at remembering to take the pill (i.e., unintentionally non‐adhering) and randomised them to one of four conditions and measured their adherence over 6 weeks. Each condition provided specific behaviour change techniques (Abraham & Michie, 2008; Michie et al., 2011) based on the behavioural mechanisms of change of the key variables (i.e., habit strength and education/knowledge). The design of this study is based on a recent successful experiment that aimed to increase the use of reusable coffee cups (Novoradovskaya et al., 2020), which can also be considered a distal reward behaviour (i.e., the reward is in the future rather than immediate; Mullan & Novoradovskaya, 2018) like that of taking the pill. The four different conditions in the experiment are: (i) control group, (ii) information only group, (iii) cue implementation only group, and (iv) information + cue implementation group.

Subsequently, it was hypothesised:

1. Based on research suggesting that habit strength is associated with better adherence (Liddelow et al., 2022), and with the assumption that our sample who self‐identifies as not being very good at remembering to take the pill (i.e., unlikely to have a high habit strength for the behaviour), there will be a positive association between pill habit strength and adherence at follow‐up.
2. Based on research that suggests education/knowledge alone is not enough to change behaviour (Arlinghaus & Johnston, 2018), participants who match their pill‐taking behaviour with their choice of a daily cue (cue implementation only group, and combined group) will report a greater increase in habit strength and adherence over time compared to the groups without a cue (control group, and information only group).
3. It is expected that at the end of 6 weeks, there will have been an increase in the primary outcome variable (adherence to the pill) and the secondary outcome variable (habit) in all three experimental conditions, but not the control group.

## METHODS

### Experimental conditions

#### Group 1–control

The control group were provided with no experimental content and were simply instructed to take their pill at the same time every day as per the usual instructions given by their health professional.

#### Group 2–information only

The information only group was provided with a standardized and general Consumer Medicine Information leaflet (see Data S1 and S3) from a credible and common pharmaceutical company in Australia, much like the ones already provided in the packet/box. These leaflets provide the consumer with information regarding how the pill works, how to take it correctly, any potential side effects, and what to do if a dose is missed. A Consumer Medicine Information leaflet was provided for both the combination pill and the progestogen‐only pill (mini‐pill), and participants randomized to this group were instructed to select the one that matched their current pill type to ensure they were receiving the correct information. Participants were instructed to try and take their pill for the next 6 weeks according to the information in the leaflet. The content provided to this group (as part of the leaflet) used three behaviour change techniques, coded in the behaviour change technique taxonomy as ‘4.1 instruction on how to perform the behaviour’, ‘5.1 information about health consequences’, and ‘9.1 credible source’ (Michie et al., 2013).

#### Group 3–cue implementation only

Participants in this group were provided with a definition of a cue and asked to spend a few minutes thinking of a cue they can match with taking their pill to assist them in remembering each day. Participants were told the cue must be something they do every day (i.e., an action‐based cue) and were provided with examples, such as ‘placing your pill box on your bedside table so you see it every morning when you wake up, acting as a reminder to take your pill’. Participants were instructed to write down their chosen cue and ensure they take their pill when they experience this cue, in the chosen context, for the next 6 weeks. The content provided used three behaviour change techniques, coded in the behaviour change technique taxonomy as ‘7.1 prompts/cues’, ‘8.1 behavioural practice/rehearsal’, and ‘8.3 habit formation’ (Michie et al., 2013).

#### Group 4–information + cue implementation

Participants in the combined information and cue implementation group received the same material from groups 2 and 3. Participants were instructed to read the general Consumer Medicine Information leaflet for their specific pill (i.e., either the Mini‐pill or combination pill) and asked to choose a cue to assist them to take their pill every day (preferably at the same time). The order the content was presented to participants was not randomized (see Data S2 for detailed instructions provided to each experimental group). The content provided to this group used the six behaviour change techniques mentioned previously.

### Participants

An *a*‐priori power analysis was conducted in G*Power (Faul et al., 2007) to see the minimum number of participants needed to detect a small‐medium effect size (*f* = 0.25; based on meta‐analysis findings from Conn and Ruppar (2017)) using a repeated measures within‐between ANOVA, with *α* = .05, power = 0.80, four groups and two‐time points, a minimum sample size of 48 (12 per group) was needed. However, accounting for at least 30% attrition, we aimed to recruit 80 participants in total (20 per group). Participants were required to be living in Australia, between the ages of 16 and 35 years (i.e., peak reproductive years for most women), currently taking the pill for contraception (prevention of pregnancy) and self‐perceived themselves as not very good at remembering to take the pill (i.e., unintentionally non‐adherent).

### Measures

#### Adherence to the pill

Adherence to the pill at both baseline and follow‐up was measured using two different self‐report measures, as previous research has identified different measures of adherence measure different things, such as attitudes/beliefs or actual behaviour, and thus perform differently in research (Liddelow et al., 2021).

##### Medication adherence report scale (Chan et al., 2020; Horne & Weinman, 1999)

The five‐item Medication Adherence Report Scale is a widely used measure of adherence. It contains five questions related to medication‐taking over the previous 2 weeks (as is typical for this measure), that are summed. For example, “*I forgot to take a dose of my pill*”, all of which are answered on a 5‐point Likert scale ranging from 1 = never to 5 = always. All items are reversed scored such that higher scores indicate better adherence. This measure showed adequate internal consistency in this sample (*α* = .71 at follow‐up to .74 at baseline).

##### Timeline follow‐back (Sobell & Sobell, 1992)

The second measure of adherence was an adapted version of the Timeline Follow‐Back which has previously been used in studies of medication adherence (Liddelow et al., 2021). This measure of adherence is presented in a calendar‐like form to prompt participants to recall their previous week and to think about each day individually. Participants are instructed to enter the day and date, starting from the day before and working backwards. They are also asked to enter any special events that may have occurred on these individual days. The next two parts ask participants to indicate whether they took their pill on that day by entering a Y (for yes) or an N (for no). The final row of the measure asks participants if, on that day, they took their pill as prescribed (typically, at the same time every day) by entering a Y or N. If participants did not take their pill that day, a response of N is expected. Participants received a total adherence score out of 14 (one score for each day taking the pill and another score if the pill was taken correctly), with higher scores indicating better adherence over the previous week. If participants did not take their pill on an individual day, they automatically got zero for that day. A timeframe of 1 week is used for this measure as this is how the measure has typically been applied in previous medication adherence‐related research (Liddelow et al., 2021).

#### Habit

Habit strength was measured using the Self‐Report Habit Index (Verplanken & Orbell, 2003). This measure is the most widely used measure of habit strength and consists of the prompt “*Ensuring I take my oral contraceptive pill correctly is something…*” followed by 12 statements such as “…I do frequently” and “I do without thinking”. Participants were asked to select how much they agree or disagree with each statement on a 7‐point Likert scale from 1 = strongly disagree to 7 = strongly agree. The scores from each item were combined and the mean score calculated. Higher mean scores indicated a higher habit strength for taking the pill. This measure showed good to excellent internal consistency in this sample (*α* = .89 at baseline to .93 at follow‐up).

#### Demographics

Participants were asked to complete 11 demographic questions, most of which related to which type of pill they were currently taking, how long they have been on the pill, whether they were sexually active, had previous pregnancies and/or children, and their use of emergency contraception. General demographics such as age, gender identity, education and ethnicity were also collected.

### Procedure

Advertisements for participation in the study were placed on social media sites such as Facebook (i.e., public Australian‐based groups such as community/neighbourhood groups), Twitter, and Reddit (i.e., research forums), between May and September 2020. Advertisements consisted of a digital poster and a brief paragraph outlining the purpose of the study and inclusion criteria (e.g., are you a woman below the age of 35 and currently take the oral contraceptive pill for contraception but are not very good at remembering to take it?), accompanied by a link for participants to click if they required more information or were interested in participating. If interested, participants were directed to an online Qualtrics survey where they were presented with the participation information sheet. This sheet did not detail the content of the four groups, meaning participants were blind to the specific experimental groups prior to being randomized. After reading the participant information sheet, participants were asked to give informed consent by checking a box. If they did not check this box, they were unable to continue the study and were thanked for their time.

Upon providing informed consent, participants entered their email address. and created a unique code to assist in linking their baseline and follow‐up responses. After this, participants were screened against the key inclusion criteria and asked to report their age (in years), whether they currently take the oral contraceptive pill, and whether they take the pill mainly for contraception (rather than other gynaecological issues). Eligible participants then completed all baseline measures, which took no longer than 20 minutes, and were then randomly assigned to one of the four groups using the built‐in random number generator setting in Qualtrics. The Qualtrics randomiser setting monitors the randomisation to ensure participants are allocated based on a 1:1 ratio, but in a random order. Participants in the combined information + cue implementation group were asked to read the Consumer Medicine Information leaflet and then to spend a few minutes thinking about a cue. This was presented in the same order each time.

After completing the baseline survey and being randomized, participants were instructed to follow the advice they had been given for the next 6 weeks. After the 6 weeks had elapsed, participants were sent an email asking them to complete the follow‐up, with a link to the online Qualtrics survey (same measures as baseline). No reminders were sent to participants after this initial email. At no stage were participants debriefed or provided information relating to the other experimental groups. Participants who fully completed both parts were placed in a prize draw to win 1 of 5 shopping vouchers valued at $50 AUD each. The Curtin University Human Research Ethics Committee approved the study (2020–0158).

### Data analysis

The data collected at both time points were combined and analysed using SPSS (IBM Corp, 2021). To ensure the data was appropriate for the planned main analyses (i.e., mixed‐model ANOVAs), several assumptions were checked prior to running any analyses. All assumptions were met, except for normality for both measures of adherence (i.e., Shapiro–Wilk test was *p* < .001 for both measures of adherence at both timepoints). Boxplots also indicated the presence of extreme outliers on both measures of adherence. Subsequently, cases identified as being extreme outliers were removed from the analysis (*n* = 4), however, it did not significantly improve the normality of the data. Both log and square root transformations of the two adherence variables was also trialled, but both also did not significantly improve the data. It is not uncommon for adherence assessed by self‐report measures to be typically highly skewed towards full adherence (e.g., Kleppe et al., 2015; Tommelein et al., 2014). Currently, there is no agreed‐upon way to overcome this limitation of the research area and therefore we continued with the planned analyses, with the extreme outliers excluded. To explore possible differences in key baseline demographics (age, type of pill, length of time using, number of children and education level) and the outcome variables (adherence and habit strength) between those who completed both time points and those who did not, Chi‐Square Test of Contingencies and independent samples *t*‐tests were used. To investigate whether randomisation was successful, a one‐way Analysis of Variance (ANOVA) was conducted on the same key demographic and outcome variables. Spearman rank correlation coefficients (*ρ*) were conducted to assess the relationships between key demographic variables (age, type of pill, length of time using, number of children and education level) and the primary outcome (adherence) and the secondary outcome (habit strength) variables at both baseline and follow‐up.

For the main analysis, three mixed‐model ANOVAs were used to test for changes in the primary (adherence) and secondary (habit strength) outcomes over time. The experimental group (i.e., control, information only, cue implementation only, and information + cue implementation) was the between‐subjects factor, with participants randomised to only one of the four conditions. Time (baseline and follow‐up scores on the primary and secondary outcomes) was the repeated measures factor. The group x time interaction tested if there were any condition‐related differences on the primary and secondary outcomes over time. If required, for significant group x time interactions, appropriate planned comparisons tested differences between all four conditions at the two‐time points, as well as differences over time within each condition.

## RESULTS

### Descriptive statistics

**FIGURE 1:**
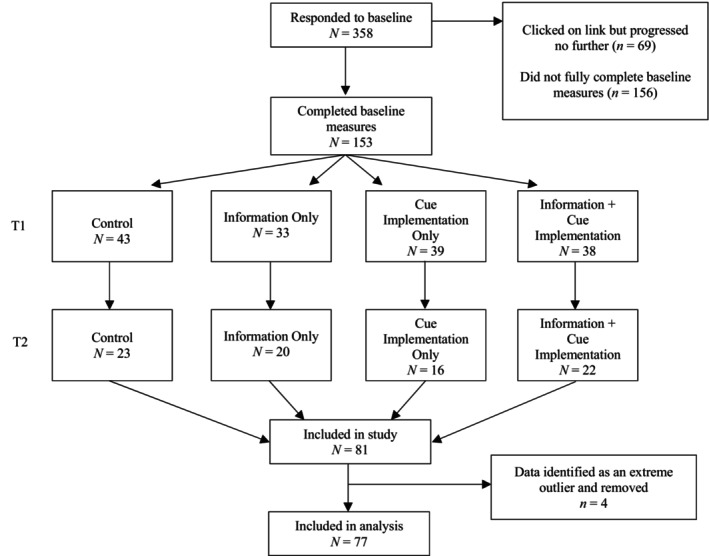
Flow Chart of Participants.

Initially, 358 participants responded to the advertising material, however, 225 of these did not complete the baseline questionnaire and thus were not randomized to a group. This left a total of 153 participants who completed the baseline questionnaire and were randomized to a group. A total of 72 participants did not complete the follow‐up, with two of these not supplying an email address to be contacted to receive the link to the follow‐up. A total of 81 participants completed both time points, however, 4 participants had their data removed due to being extreme outliers. This left a total of 77 participants whose data was subsequently used in the analysis (see Figure 1 for a flow chart of participants). There were no significant differences between completers of both time points and non‐completers on any demographics or key variables at baseline assessment.

Of those that completed both parts (*N* = 77), all participants identified as biologically female and their ages ranged between 18 and 34 years (*M*_age_ = 25.18, *SD* = 3.49). The sample was highly educated with 72.7% (*n* = 56) having a bachelor's degree or higher. A total of 87.0% (*n* = 67) of the sample identified as Caucasian. The majority 83.1% (*n* = 64) reported currently using the combined pill (one‐month cycle), 11.7% (*n* = 9) using the combined pill (13 weeks cycle) and the remaining 5.2% (*n* = 4) were currently using the progestin‐only pill, or the ‘mini‐pill’. The length of time taking the pill ranged from 2 months to 16 years (*M* = 7.13 years, *SD* = 4.18), with 67.5% (*n* = 52) having been recommended the pill by their General Physician or Gynaecologist. Only 7.8% (*n* = 6) of participants had children and 18.2% (*n* = 14) reported ever being pregnant. Just over half of the sample, 51.9% (*n* = 40) reported having ever used emergency contraception. See Table 1 for the baseline demographic characteristics of each group and overall.

**TABLE 1:** Baseline demographic characteristics between groups and overall.

	Control (*n* = 21)	Information only (*n* = 19)	Cue implementation only (*n* = 16)	Information + Cue implementation (*n* = 21)	Total (*N* = 77)
	Mean ± *SD*/*n* (% group)	Mean ± *SD*/*n* (% group)	Mean ± *SD*/*n* (% group)	Mean ± *SD*/*n* (% group)	Mean ± *SD*/*n* (% group)
Age	25.67 ± 3.92	24.89 ± 3.04	26.44 ± 3.83	24.00 ± 2.93	25.18 ± 3.49
Type of pill					
Combination	20 (95.2)	17 (85.5)	15 (93.8)	22 (100.0)	73 (94.8)
Mini‐pill	1 (4.8)	2 (10.5)	1 (6.3)	0 (0.0)	4 (5.2)
Education					
≤Secondary school	2 (9.5)	7 (36.8)	5 (31.3)	7 (33.3)	21 (27.3)
Bachelor's degree	15 (71.4)	7 (36.8)	10 (62.5)	12 (57.1)	44 (57.1)
≥Master's degree	4 (19.0)	5 (26.3)	1 (6.3)	2 (9.5)	12 (15.6)
Ethnicity					
Caucasian/European	18 (85.7)	16 (84.2)	15 (93.8)	18 (85.7)	67 (87.0)
Asian/Middle Eastern	3 (14.3)	3 (15.8)	1 (6.3)	2 (9.5)	9 (11.7)
Indigenous	0 (0.0)	0 (0.0)	0 (0.0)	1 (4.8)	1 (1.3)

Abbreviations: MARS‐5, Medication Adherence Report Scale; *SD*, standard deviation; TLFB, Timeline Follow‐Back.

Overall, participants reported reasonably high adherence at baseline, particularly when using the Medication Adherence Report Scale (MARS‐5) with *M* = 22.91 out of a possible 25. Adherence, as measured by the Timeline Follow‐Back, indicates that, on average, participants are adherent between 4 and 5 days a week. However, habit strength overall was average with *M* = 3.93 out of a possible 7.

### Randomisation check

We compared the means of key variables at baseline for each of the four different groups using a one‐way ANOVA. No significant differences between groups were identified on age, number of children, habit strength, or either of the measures of adherence to the pill. We also compared differences in categorical demographic variables (type of pill, ethnicity, and education level) and found no significant differences between the groups.

### Associations between variables

Spearman Rank correlations were computed to assess the strength of the association between the main variables of interest (see Table 2). No demographic variables were significantly correlated with adherence or habit strength at both baseline or follow‐up. At follow‐up, there were significant positive correlations between habit strength for taking the pill and adherence using both measures; Medication Adherence Report Scale (*ρ* = .481, *p* < .001) and the Timeline Follow‐Back (*ρ* = .452, *p* < .001).

**TABLE 2:** Spearman rank correlations (*ρ*) at Baseline (T1) and Follow‐up (T2).

	Age	Type of pill	Length of time using	Number of children	Ethnicity	Education level	MARS‐5 T1	TLFB T1	Habit T1	MARS‐5 T2	TLFB T2	Habit T2
Age	–											
Type of Pill	.045	–										
Length of time using	.520**	−.040	–									
Number of Children	.319**	−.068	.379**	–								
Ethnicity	.141	.081	−.303**	−.112	–							
Education Level	.501**	.053	.175	−.171	.133	–						
MARS‐5 T1	.057	.124	−.129	.122	.011	.015	–					
TLFB T1	.145	.181	−.039	.113	.050	−.156	.406**	–				
Habit T1	−.053	.213	−.037	.149	.020	−.051	.363**	.279*	–			
MARS‐5 T2	.006	.062	−.112	.202	.106	.043	.481**	.228*	.312**	–		
TLFB T2	.065	−.068	−.104	.026	.160	−.029	.149	.270*	.272**	.391**	–	
Habit T2	−.039	.109	−.069	.215	.102	−.074	.264*	.209	.615**	.481**	.452**	–

*Note*: Type of Pill (1 = combination pill, 2 = mini‐pill), Ethnicity (1 = Caucasian/European, 2 = Asian/Middle Eastern, 3 = Aboriginal or Torres Strait Islander), Education Level (1 = High school certificate or below, 2 = More than high school certificate).

Abbreviations: MARS‐5, Medication Adherence Report Scale; TLFB, Timeline Follow Back.

**p < .001.

*p < .05.

### Effects of the experiment: mixed model ANOVAs

A series of 4 (experimental condition) x 2 (time) mixed‐model ANOVAs were conducted to assess the change in adherence to the pill (using both measures), and habit strength between the four different groups over time (see Table 3 for the mixed‐model ANOVA results).

**TABLE 3:** Results of the Mixed‐Model ANOVA for Each Outcome Variable.

Outcome	*F*	*Df*	*p*‐value	Partial eta squared
MARS‐5				
Main effects				
Time	0.00	1	.991	0.000
Group	1.01	3	.395	0.040
Interaction				
Time x Group	1.87	3	.143	0.071
TLFB				
Main effects				
Time	0.38	1	.542	0.006
Group	1.32	3	.275	0.060
Interaction				
Time x Group	2.19	3	.098	0.096
Habit strength				
Main effects				
Time	8.98	1	.004*	0.110
Group	2.88	3	.042	0.106
Interaction				
Time x Group	2.44	3	.071	0.091

Abbreviations: MARS‐5, Medication Adherence Report Scale; TLFB, Timeline Follow‐Back.

*p < .05.

#### Primary outcome

##### Adherence: medication adherence report scale (MARS‐5)

A significant main effect for time was not identified *F*(1, 73) = 0.00, *p* = .991, partial *η*2 = .00 meaning there was no change in adherence to the pill over time using this measure. There was also no significant main effect of group identified *F*(3, 73) = 1.01, *p* = .395, partial *η*2 = .04, and no significant interaction between time and group *F*(3, 73) = 1.87, *p* = .143, partial *η*2 = .07. This suggests that adherence to the pill did not significantly increase in all groups over time.

##### Adherence: timeline follow‐back (TLFB)

The main effect of time was not significant *F*(1, 62) = 0.38, *p* = .542, partial *η*2 = .01, and a significant main effect for group was also not identified *F*(3, 62) = 1.32, *p* = .275, partial *η*2 = .06. A non‐significant interaction between time and group was also reported *F*(3, 62) = 2.19, *p* = .098, partial *η*2 = .10, such that adherence to the pill did not significantly increase in all groups over time.

#### Secondary outcome

##### Habit strength

A significant main effect for time was identified *F*(1, 73) = 8.99, *p* = .004, partial *η*2 = .11. A significant main effect for group was also identified *F*(3, 73) = 2.88, *p* = .042, partial *η*2 = .11. However, a significant interaction between time and group was not identified, *F*(3, 73) = 2.44, *p* = .071, partial *η*2 = .09, such that habit strength significantly increased over time across all groups, but the changes over time did not significantly differ across the groups. Examination of the means indicated that the information only group reported the largest change in habit strength from baseline (*M* = 4.07, *SD* = 1.01) to follow‐up (*M* = 4.99, *SD* = 1.37). The control group reported the smallest change in habit strength from baseline (*M* = 3.72, *SD* = 1.14) to follow‐up (*M* = 3.79, *SD* = 1.01). See Table 4 for the descriptive statistics of each group at both time points for all the outcome variables.

**TABLE 4:** Descriptive statistics of the outcome variables for each group at both timepoints.

Variable	Time	Control (*n* = 21)	Information only (*n* = 19)	Cue implementation only (*n* = 16)	Information + Cue implementation (*n* = 21)	Total (*N* = 77)
		Mean ± *SD*	Mean ± *SD*	Mean ± *SD*	Mean ± *SD*	Mean ± *SD*
MARS‐5 (2 weeks)	Baseline	22.67 ± 1.83	23.21 ± 1.69	22.88 ± 2.63	22.90 ± 2.51	22.91 ± 2.15
	Follow‐up	23.24 ± 1.55	23.47 ± 2.04	21.63 ± 3.80	23.33 ± 2.27	22.99 ± 2.51
TLFB (1 week)	Baseline	10.35 ± 3.91	11.94 ± 2.72	12.27 ± 2.05	10.05 ± 4.43	10.97 ± 3.63
	Follow‐up	11.45 ± 2.31	12.88 ± 1.67	9.91 ± 5.22	11.74 ± 3.38	11.62 ± 3.23
Habit strength	Baseline	3.72 ± 1.14	4.07 ± 1.01	3.66 ± 1.03	4.23 ± 1.09	3.93 ± 1.09
	Follow‐up	3.79 ± 1.01	4.99 ± 1.37	3.90 ± 1.11	4.46 ± 1.29	4.29 ± 1.28

Abbreviations: MARS‐5, Medication Adherence Report Scale; *SD*, standard deviation; TLFB, Timeline Follow‐Back.

### Sensitivity analysis

To explore whether findings related to habit stays true when using a different measure of habit, the Self‐Report Behavioural Automaticity Index (SRBAI; Gardner et al., 2012), the mixed‐model ANOVA analyses were repeated. This measure contains four items from the original Self‐Report Habit Index, all of which focus on the automaticity of habit (rather than the frequency). The items are measured the same as in the original measure.

A 4 (experimental condition) x 2 (time) mixed‐model ANOVA was conducted to assess the change in SRBAI habit strength between the four different groups over time. A significant effect for time was identified *F*(1, 73) = 8.75, *p* = .004, partial *η*2 = .11. No significant main effect for group was identified *F*(3, 73) = 2.18, *p* = .082, partial *η*2 = .08. In addition, no significant main effect for the interaction between time and group was identified, *F*(3, 73) = 1.77, *p* = .160, *η*2 = .068 suggesting that automaticity significantly increased over time across all groups, but the changes over time did not significantly differ between the groups.

## DISCUSSION

A six‐week repeated‐measures experiment was conducted to improve adherence to the pill. Two different mechanisms, namely cues and information from a credible source, were hypothesised to improve medication adherence and habit strength. The findings supported our first hypothesis, with habit strength and adherence being significantly positively associated at follow‐up. Hypotheses two was not supported as all experimental groups, including those that did not receive the cue implementation, increased their habit strength over time, and there were no significant differences in this increase between the groups. Finally, hypothesis three was not supported as there were no significant increases in the primary outcome (adherence to the pill) between the three experimental groups over time. Contrary to our hypothesis, habit strength increased across all groups (including the control group) over time.

There were no significant effects identified on either measure of the primary outcome of medication adherence. This suggests the experiment was unsuccessful in changing behaviour. One possibility for this is the chosen behaviour was not a novel behaviour for the participants and thus it was more difficult to elicit changes in behaviour as participants were already in their own routines (Mergelsberg et al., 2020). This aligns with experimental cue implementation studies that found significant improvements in behaviour when the behaviour was novel, such as microwaving a dishcloth to reduce cross‐contamination (Keller et al., 2021; Mergelsberg et al., 2020). Another possible reason why the experiment was unsuccessful in changing adherence behaviour was while sufficiently powered based on the power analysis, it may have been underpowered to detect any significant effects given the ceiling effect in both adherence measures. This lack of variation in adherence is not uncommon, with several studies reporting the same issue yet for other medications (Kleppe et al., 2015; Tommelein et al., 2014). Therefore, replicating the experiment with population of less adherent participants is warranted to either prove or disprove the current findings. It is also possible that participants in the study believed they were poor at adhering to the pill for reasons that were not addressed in the experiment. For example, participants may have had issues with motivation (e.g., not having regular intercourse so did not see the purpose of taking the pill) that influenced their unintentional adherence. As such, participating in this study and receiving any of the conditions was unlikely to improve their behaviour as their main barrier was not addressed. Research shows that motivational barriers need to be addressed before adherence or habit strength will improve (Horne & Weinman, 1999; Verplanken & Wood, 2006). Nonetheless, full adherence to the pill is still required to ensure the greatest benefits and protection against unintentional pregnancy (Teal & Edelman, 2021), and further studies addressing ways to improve adherence are still required.

Neither the use of a cue nor providing information was able to significantly change behaviour. Common behaviour change techniques aimed to change behaviour were included, however, only changes in the secondary outcome, habit, were identified. One possible explanation for why cues did not elicit significant changes in behaviour may be due to the types of cues participants selected to use. Although participants were not required to report the cue they chose, the example in the instructions provided to participants stated that the cue was ‘to be something they do every day’ (i.e., an action‐based cue). This recommendation was based on previous research by Stawarz et al. (2016) who found that contextual cues, such as routine events, locations and meaningful objects, were more effective than time‐based cues. However, it may be possible that for this specific group, contextual cues are not effective and other types of cues may be (e.g., time‐based, sensory‐based). However, future research is needed to compare the effects of using various types of cues in improving adherence behaviours. Nonetheless, this is an interesting finding and suggests that habit may be an underlying mechanism for adherence to the oral contraceptive pill. This aligns with previous research that has shown habit strength is a predictor of adherence to medications more broadly (Liddelow et al., 2022; Phillips et al., 2013). It also suggests that cue implementation and information from credible sources may provide a basis for strengthening habit rather than for strengthening behavioural engagement.

The findings showed a significant positive association between habit strength for taking the pill at follow‐up and adherence using both measures. This is consistent with recent research which has found an association between habit and medication adherence generally (Badawy et al., 2020) and more specifically to the pill (Murphy et al., 2018). Furthermore, it was demonstrated that all groups experienced an increase in their habit strength from baseline to follow‐up, which has also been shown in other studies that use mechanisms of habit formation (Bartle et al., 2019; Rompotis et al., 2014). This finding suggests that simply participating in an experiment can increase good habits, regardless of the condition.

Interestingly, all groups in the experiment, including the control group, reported increases in their habit strength. However, no group saw greater increases than another. This suggests that providing non‐habit‐based content still has the capacity to strengthen habit (Gardner et al., 2023). However, our sample was highly educated and thus likely to have higher levels of health literacy (Adams et al., 2009; Heijmans et al., 2015), which is the ability to find, understand and use health information (Sørensen et al., 2012). Therefore, it appears that in a highly educated population, providing information from credible sources can increase habit strength for a healthy behaviour, but not actually increase engagement in the behaviour. Studies for similar behaviours should therefore consider the use of fact sheets and if medication‐related, Consumer Medicine Information leaflets.

Furthermore, habit strength increased across all conditions, including the control group. This finding is contrary to what was hypothesised as changes in habit strength were only expected in the experimental groups. The control group were instructed to ‘take their pill at the same time every day as per the usual instructions given by their health professional’. Despite this instruction likely being the same as what they were told when first prescribed the pill (i.e., treatment as usual), perhaps this instruction and re‐iteration of what is already known acted as a reminder to participants and thus yielded some change in the habitual behaviour of the participants. Future research may consider being more subjective with the instructions provided to the control group/s. However, despite these changes in habit strength, they did not translate into behaviour change. One possibility is that although habit strength increased, it may not have been enough for the behaviour to become ‘habitual’ and thus actually influenced by habit. According to Lally and colleagues (Lally et al., 2010), a habit can take anywhere from 18 to 254 days to form, and a more recent study found it takes a median of 59 days (8 weeks) for a behaviour to reach peak automaticity (Keller et al., 2021). The findings of the sensitivity analysis using the four‐item Self‐Report Behavioural Automaticity Index (Gardner et al., 2012) support this finding by suggesting that automaticity also did not improve in any groups over time. As the pill is a distal benefit behaviour and the benefits of taking it are not experienced immediately (Mullan & Novoradovskaya, 2018), it may take longer for the behaviour to become habitual. Future research should consider extending the experimental period from 6 weeks to perhaps 24 weeks, to explore whether this increased measurement time sees more significant changes in habit strength, specifically automaticity, and subsequently adherence to the pill. Conversely, given that participants were already taking the pill for some time, they likely already had some sort of routine for taking the pill, and thus a degree of ‘habit’, which may have been disrupted by this study. According to Graybiel and Smith (2014) whilst one habit may be able to replace another, the initial habit can never be truly ‘broken’. Therefore, future research may consider targeting those who are only just beginning to use the oral contraceptive pill or asking participants to identify their current pill‐taking habit and focus on a way to strengthen it (i.e., setting a timer to ensure they follow through with taking the pill after their evening meal).

### Limitations

The study is not without its limitations. Firstly, adherence at baseline (and follow‐up) in all groups was reasonably highly skewed and this may explain the lack of significant effects; there was not enough room for positive improvement, despite the need for full adherence when taking the pill. Studies in the future should consider other avenues for recruiting participants that may not be as good at adhering to their medication or are only just beginning to use the pill. Secondly, the use of self‐report measures, especially for variables that are deemed to occur in the unconscious, such as habit (Hagger et al., 2015; Sniehotta & Presseau, 2012), has been contested (Gardner et al., 2023) and therefore the accuracy of the results may be limited. Similarly, self‐report medication adherence measures may be open to reporting and social biases (Stirratt et al., 2015), and are often met with highly positively skewed results (Kleppe et al., 2015), but they were the optimal choice for this study given its online nature, their ease of use, and the ability to collect data without being face‐to‐face. The findings also need to be interpreted with caution given the skewness of the outcome variables. Thirdly, including a measure of oral contraceptive pill knowledge at each time point, or recording the amount of time participants spent reading the leaflet, may have given additional insight into the mechanisms of change within the experiment. It would have also highlighted whether participants read and/or engaged with the Consumer Medicine Information leaflet. Similarly, the information only group was provided a leaflet that is already included with many prescription medications in Australia. As such, this group could be considered to be a second control group rather than an experimental group. However, future research is needed to truly understand the rates at which these leaflets are read/engaged with as whilst it may be provided, it may not be read. Finally, participants were not asked to report the cue that they chose, meaning we were unable to examine whether participants (i) understood the instructions correctly, or (ii) selected an appropriate cue. Therefore, it is unknown whether participants in the Cue Implementation Only and the Information + Cue Implementation group effectively engaged in a cue‐based approach. It is recommended that future research has participants report their cue to ensure instructions were followed as intended and consider providing alternative examples of action‐based cues in the instructions to participants to ensure they fully understand the types of cues they can use.

## CONCLUSION

The current study was unsuccessful in eliciting changes in adherence to the pill, such that providing information from a credible source nor associating taking the pill with a daily cue substantially increased adherence. However, all groups in the experiment, including the control group, reported increases in their habit strength. However, no group saw greater increases than another. The findings suggest that none of the BCTs included in the study were successful in changing behaviour, but rather only the underlying mechanisms of the behaviour (e.g., habit). However, adherence was positively skewed and therefore the findings need to be considered with this in mind. Nonetheless, full adherence to the pill is imperative to ensure the highest effectiveness against unintended pregnancies, and therefore more studies are needed to investigate how to improve adherence to this medication.

## AUTHOR CONTRIBUTIONS

**Caitlin Liddelow:** Conceptualization; data curation; formal analysis; funding acquisition; investigation; methodology; writing – original draft; writing – review and editing. **Barbara A. Mullan:** Conceptualization; supervision; writing – review and editing. **Mark Boyes:** Conceptualization; supervision; writing – review and editing.

## ACKNOWLEDGEMENTS

Open access publishing facilitated by The University of Western Australia, as part of the Wiley ‐ The University of Western Australia agreement via the Council of Australian University Librarians.

## FUNDING INFORMATION

This study was funded by the Australian Government in the form of a Stipend Scholarship program for Higher Degree by Research students. Mark Boyes is supported by the National Health and Medical Research Council Australia [Investigator Grant 1173043].

## CONFLICT OF INTEREST STATEMENT

The authors report no conflicts of interest.

## Supporting information


Data S1.

Data S2.

Data S3.

## Data Availability

The participants of this study did not give written consent for their data to be shared publicly, so due to the sensitive nature of the research supporting data is not available.

## Footnotes

^1^
The term ‘perfect adherence’ is often used when describing adherence to the oral contraceptive pill (e.g., Coombe et al., 2016). However, we feel this term has a value judgement attached to it. Therefore, throughout the manuscript ‘perfect adherence’ will instead be referred to as ‘full adherence’.
