# Supplementary material for: A simple experiment to improve adherence for taking the oral contraceptive pill: An exploratory study of behavioural mechanisms
Source: Br J Health Psychol. 2025 Mar 3;30(2):e12788. doi: 10.1111/bjhp.12788 (PMC11876489; doi:10.1111/bjhp.12788)
Supplement: Supplementary file 1 — Data S1. [file BJHP-30-0-s001.pdf]

# LEVLEN® ED

## (Lev-len Ee-Dee)

Contraceptive tablets for women

*ethinylestradiol and levonorgestrel*

## Consumer Medicine Information

## WHAT IS IN THIS LEAFLET

This leaflet answers some common questions about Levlen ED. It does not contain all the available information. It does not take the place of talking to your doctor or pharmacist.

All medicines have risks and benefits. Your doctor has weighed the risks of you taking Levlen ED against the benefits they expect it will have for you.

**If you have any concerns, or are unsure about taking this medicine, ask your doctor or pharmacist for more advice.**

**Keep this leaflet with the medicine.**
You may need to read it again.

## WHAT LEVLEN ED IS USED FOR

Levlen ED is a combined oral contraceptive, commonly known as a 'birth control pill' or 'the Pill'.

Levlen ED is used to prevent pregnancy.

You may also experience the following benefits:

- more regular and lighter periods – potentially resulting in a decrease in anaemia (iron deficiency)
- a decrease in period pain.

Some conditions such as pelvic inflammatory disease, ovarian cysts, ectopic pregnancy (where the foetus is carried outside of your womb), lumpy breasts, acne and cancer of the uterus (womb) and ovaries may be less common in women taking the Pill.

When taken correctly, Levlen ED prevents you from becoming pregnant in several ways, including:

- inhibiting ovulation (egg release)
- changing the cervical mucus consistency, making it more difficult for the sperm to reach the egg
- changing the lining of the uterus, making it less suitable for implantation.

When the Pill is taken by women under close observation in clinical trials, it is more than 99% effective in preventing pregnancy. However, in real life the Pill is around 92% effective. This is because pills might have been missed, may have been taken with medicines that interfere with their effectiveness, or may not be absorbed due to vomiting or diarrhoea.

Like all oral contraceptives, Levlen ED is intended to prevent pregnancy. It does not protect against HIV infection (AIDS) and other sexually transmitted infections.

**Ask your doctor if you have any questions about why this medicine has been prescribed for you.**
Your doctor may have prescribed it for another reason.

## BEFORE YOU TAKE LEVLEN ED

### *When you must not take it*

**Do not take Levlen ED if you have an allergy to:**

- ethinylestradiol and/or levonorgestrel (the active ingredients in Levlen ED)
- any of the ingredients listed at the end of this leaflet.

Some of the symptoms of an allergic reaction may include:

- shortness of breath
- wheezing or difficulty in breathing
- swelling of the face, lips, tongue or other parts of the body
- rash, itching or hives on the skin.

**Do not take Levlen ED if you have or have had a blood clot in:**

- the blood vessels of the legs (deep vein thrombosis - DVT)
- the lungs (pulmonary embolism - PE)

- the heart (heart attack)
- the brain (stroke)
- other parts of the body.

**Do not take Levlen ED if you have or are concerned about an increased risk of blood clots.**
Blood clots are rare. Very occasionally blood clots may cause serious permanent disability, and may even be fatal.

You are more at risk of having a blood clot when you take the Pill. However, the risk of having a blood clot when taking the Pill is less than the risk of having a blood clot during pregnancy.

**Do not take Levlen ED if you are concerned about an increased risk of blood clots because of age or smoking.**
The risk of having a heart attack or stroke increases as you get older. It also increases if you smoke.
You should stop smoking when taking the Pill, especially if you are older than 35 years of age.

**Do not take Levlen ED if you are taking any antiviral medicines which contain ombitasvir, paritaprevir and/or dasabuvir.**
These antiviral medicines are used to treat chronic (long-term) hepatitis C (an infectious disease that affects the liver, caused by the hepatitis C virus (HCV)).

**Do not take Levlen ED if you have, or have had:**

- any blood clotting disorders such as Protein C deficiency, Protein S deficiency, Leiden Factor V mutation, Antithrombin III deficiency or other inherited blood clotting conditions
- a confirmed blood test showing:
    - increased levels of homocysteine
    - antiphospholipid antibodies (APLAs) e.g. anticardiolipin-antibodies and lupus anticoagulant. These may increase your risk for blood clots or pregnancy losses (miscarriage)
- major surgery after which you have not been able to move around for a period of time
- angina (chest pain)
- a mini-stroke (also known as TIA or transient ischaemic attack)
- migraine, where you have also had problems with seeing, speaking or had, or weakness or numbness in any part of your body
- high risk of blood clots due to conditions such as diabetes with blood vessel damage, severe high blood pressure or severe high or low level of fats in your blood
- pancreatitis (an inflammation of the pancreas) associated with high levels of fatty substances in your blood
- severe liver disease and your liver function has not returned to normal
- cancer that may grow under the influence of sex hormones (e.g. of the breast or the genital organs)
- a benign or malignant liver tumour
- unexplained vaginal bleeding.

**If any of these conditions appear for the first time while using the Pill, stop taking it at once and tell your doctor. In the meantime, use non-hormonal (barrier) methods of contraception (such as condoms or a diaphragm).**

**Do not take this medicine if you are pregnant or think you might be pregnant.**

**Do not give this medicine to a child.**
Levlen ED is not intended for use in females whose periods have not yet started.

**Do not take this medicine after the expiry date printed on the pack and blister.**
The expiry date is printed on the carton and on each blister after "EXP" (e.g. 11 18 refers to November 2018). The expiry date refers to the last day of that month. If it has expired return it to your pharmacist for disposal.

**Do not take this medicine if the packaging is torn or shows signs of tampering.**
If the packaging is damaged, return it to your pharmacist for disposal.

**If you are not sure whether you should start taking this medicine, talk to your doctor.**

### *Before you start to take it*

**Tell your doctor if you have allergies to any other medicines, foods, preservatives or dyes.**
**Tell your doctor if:**

- you smoke
- you or anyone in your immediate family has had blood clots in the legs (DVT), or lungs (PE), a heart attack, a stroke, breast cancer or high cholesterol.

**Tell your doctor if you have, or have had any of the following medical conditions:**

- diabetes
- high blood pressure
- heart valve disorders or certain heart rhythm disorders
- migraine
- cancer
- hyperhomocysteinaemia, a condition characterised by high levels of the amino acid homocysteine in the blood
- high or low level of fats in your blood.

**Ask your doctor to check if you:**

- are overweight
- have any hereditary or acquired conditions that may make it more likely for you to get blood clots
- have high cholesterol or triglycerides
- have liver disease
- have gall bladder disease
- have jaundice (yellowing of the skin) and/or pruritus (itching of the skin) related to cholestasis (condition in which the flow of bile from the liver stops or slows)
- have Crohn's disease or ulcerative colitis (chronic inflammatory bowel disease)
- have systemic lupus erythematosus (SLE – a disease affecting the skin all over the body)
- have haemolytic uraemic syndrome (HUS – a disorder of blood coagulation causing failure of the kidneys)
- have sickle cell disease
- have a condition that occurred for the first time, or worsened during pregnancy or previous use of sex hormones (e.g. hearing loss, a metabolic disease called porphyria, a skin disease called herpes gestationis, a neurological disease called Sydenham's chorea)
- have chloasma (yellowish-brown pigmentation patches on the skin, particularly of the face) – if so, avoid exposure to the sun or ultraviolet radiation
- have hereditary angioedema – you should see your doctor immediately if you experience symptoms of angioedema, such as swollen face, tongue and/or pharynx and/or difficulty swallowing, or hives together with difficulty in breathing.

**If any of the above conditions appear for the first time, recur or worsen while taking Levlen ED, you should tell your doctor.**

**Tell your doctor if you are breastfeeding.**
Levlen ED is generally not recommended if you are breastfeeding.

**Levlen ED contains lactose.**
If you have an intolerance to some sugars, tell your doctor before you start taking Levlen ED.

**If you have not told your doctor about any of the above, tell him/her before you start taking Levlen ED.**

### *Taking other medicines*

**Tell your doctor or pharmacist if you are taking any other medicines, including any that you get without a prescription from your pharmacy, supermarket or health food shop.**

Some medicines and Levlen ED may interfere with each other. These include:

- medicines used to treat tuberculosis such as rifampicin, rifabutin
- a class of antibiotics known as macrolides, such as clarithromycin, erythromycin
- medicines used to treat fungal infections, such as ketoconazole, griseofulvin
- medicines used to treat HIV, such as ritonavir, nevirapine
- some medicines used to treat HCV, such as boceprevir, telaprevir, ombitasvir, paritaprevir, dasabuvir
- medicines used to treat epilepsy such as phenytoin, primidone, barbiturates (e.g. phenobarbitone), carbamazepine, oxcarbazepine, topiramate, felbamate, lamotrigine
- ciclosporin, an immunosuppressant medicine
- etoricoxib, a medicine used to treat painful joint disease
- melatonin, a hormone used as a sleep aid
- midazolam, a medicine used as a sedative
- theophylline, a medicine used to treat respiratory disease
- tizanidine, a medicine used as a muscle relaxant
- some medicines used to treat high blood pressure, chest pain or irregular heartbeats such as diltiazem, verapamil
- herbal medicines containing St John's Wort
- grapefruit juice.

These medicines may be affected by Levlen ED, or may affect how well it works. Your doctor may need to alter the dose of your medicine, or prescribe a different medicine.

**You may need to use additional barrier methods of contraception (such as condoms or a diaphragm) while you are taking any of these medicines with Levlen ED and for some time after stopping them.**
Your doctor will be able to tell you how long you will need to use additional contraceptive methods.

Your doctor and pharmacist have more information on medicines that you need to be careful with or avoid while taking this medicine.

## HOW TO TAKE LEVLEN ED

**Follow all directions given to you by your doctor or pharmacist carefully.** They may differ from the information contained in this leaflet.

**If you do not understand the instructions on the pack, ask**

**your doctor or pharmacist for help.**

### How to take it

Take one tablet daily at about the same time every day. You must take Levlen ED every day regardless of how often you have sex. This will also help you remember when to take it.

**Swallow the tablet whole with a full glass of water.** It does not matter if you take it before or after food.

Each blister pack is marked with the day of the week. **Take your first tablet from the red area on the blister pack corresponding to the day of the week.**

**Follow the direction of the arrows on the blister pack until all the tablets have been taken.**

A period should begin 2 to 3 days after starting to take the white inactive tablets (last row) and may not have finished before the next pack is started.

**Always start a new blister pack on the same day of the week as your previous pack.**

### Taking Levlen ED for the first time

If you are starting Levlen ED after a natural cycle, and you have not used a hormonal contraceptive in the past month, start on the first day of your period, i.e. the first day of menstrual bleeding.

**You must also use additional barrier contraceptive precautions (e.g. condoms or a cap or diaphragm with spermicide) for the first 14 days of tablet-taking when having intercourse.**

**Your doctor will advise you when to start if you:**

- are taking Levlen ED after having a baby
- have had a miscarriage or an abortion.

### Changing from another contraceptive

<u>**Changing from a combined oral contraceptive:**</u>
Start taking Levlen ED on the day after taking the last active tablet in your previous Pill pack. Bleeding may not occur until the end of the first pack of Levlen ED.

**If you are not sure which were the active/inactive tablets in your previous Pill pack, ask your doctor or pharmacist.** Your previous Pill pack may have different colour tablets to those of Levlen ED.

<u>**Changing from a vaginal ring:**</u>
Start taking Levlen ED on the day of removal of the ring but at the latest when the next application would have been due.

<u>**Changing from a progestogen-only pill ('minipill'):**</u>
Stop taking the minipill on any day and start taking Levlen ED at the same time the day after you took your last minipill.

**You must also use additional barrier contraceptive precautions (e.g. condoms or a diaphragm) for the first 14 days of tablet-taking when having intercourse.**

<u>**Changing from a progestogen-only injection, implant or intrauterine system (IUS):**</u>
Start taking Levlen ED when your next injection is due, or on the day that your implant or IUS is removed.

**You must also use additional barrier contraceptive precautions (e.g. condoms or a diaphragm) for the first 14 days of tablet-taking when having intercourse.**

### Stopping Levlen ED

You can stop taking Levlen ED at any time. If you are considering becoming pregnant, it is recommended that you begin taking a vitamin supplement containing folic acid. It is best that you start taking folic acid tablets before you stop taking Levlen ED and not stop until your doctor advises this. Ask your doctor or pharmacist about suitable supplements. It is both safe and recommended that you take folic acid during pregnancy.

### Additional contraceptive precautions

When additional contraceptive precautions are required you should either abstain from sex, or use a barrier method of contraception, a cap (or diaphragm) plus spermicide, or a condom. Rhythm methods are not advised as the Pill disrupts the cyclical changes associated with the natural menstrual cycle e.g. changes in temperature and cervical mucus.

### If you forget to take Levlen ED

If you miss a tablet and take the missed tablet within 12 hours of missing it, you should still be protected against pregnancy.

**If you are more than 12 hours late follow these detailed instructions:**

**For Levlen ED to be most effective, beige active tablets need to be taken uninterrupted for 7 days.**

**If you have been taking the beige active tablets for 7 uninterrupted days and miss a beige active tablet, take the missed tablet as soon as you remember, then go back to taking your Pill as you would normally, even if this means taking two tablets in one day, at the same time.** You should still be protected against pregnancy.

The chance of pregnancy after missing a beige active tablet depends on when you missed the tablet. **There is a higher risk of becoming pregnant if you miss a tablet at the beginning or end of a pack.**

**If after taking your missed tablet you have less than 7 days of beige active tablets left in a row, you should finish the active tablets in your pack but skip the white inactive tablets. Start taking the beige active tablets in your next pack corresponding to the correct day of the week.**

This is the best way to maintain contraceptive protection. However, you may not have a period until the end of the beige active tablets of the second pack. You may have spotting or breakthrough bleeding on tablet-taking days.

**If you have been taking the beige active tablets for less than 7 days and miss a beige active tablet, take the missed tablet as soon as you remember, then go back to taking your Pill as you would normally, even if this means taking two tablets in one day, at the same time. In addition, you must also use additional barrier contraceptive precautions (e.g. condoms or a diaphragm) for the next 7 days.**

If you have had sexual intercourse in the preceding 7 days, there is a possibility of pregnancy and you may need emergency contraception. You should discuss this with your doctor or pharmacist.

**If you forget to take more than one beige active tablet, seek advice from your doctor or pharmacist about what to do.**

If you have had sexual intercourse in the week before missing your tablets, there is a possibility of becoming pregnant.

**If you miss a white inactive tablet, you do not need to take them later because they do not contain any active ingredients.** However, it is important that you discard the missed white tablet(s) to make sure that the number of days between taking active tablets is not increased as this would increase the risk of pregnancy. Continue with the next tablet at the usual time

***Please see the table at the end of this leaflet, "Summary of advice if you missed a beige active tablet more than 12 hours ago".***

**Ask your doctor or pharmacist to answer any questions you may have.**

### *If you take too much (overdose)*

**Immediately telephone your doctor or the Poisons Information Centre (Australia: 13 11 26) for advice, or go to the Accident and Emergency Department at your nearest hospital, if you think that you or anyone else may have taken too much Levlen ED. Do this even if there are no signs of discomfort or poisoning.** You may need urgent medical attention.

## WHILE YOU ARE TAKING LEVLEN ED

### *Things you must do*

**Tell any doctors, dentists and pharmacists who treat you that you are taking this medicine.**

**If you are about to have any blood tests, tell your doctor that you are taking this medicine.** It may interfere with the results of some tests.

**Have regular check-ups with your doctor.** When you are taking the Pill, your doctor will tell you to return for regular check-ups, , including getting a Cervical Screening Test. Your doctor will advise how often you need a Cervical Screening Test. A Cervical Screening Test can detect abnormal cells lining the cervix. Sometimes abnormal cells can progress to cancer.

**If you are about to start on any new medicine, remind your doctor and pharmacist that you are taking Levlen ED.**

**Stop taking Levlen ED and see your doctor immediately if you notice the following signs:**

- one-sided swelling of the leg and/or foot or along a vein in the leg
- pain or tenderness in the leg which may be felt only when standing or walking
- increased warmth in the affected leg; red or discoloured skin on the leg
- sudden onset of unexplained shortness of breath or rapid breathing
- sudden coughing or coughing up of blood
- sharp chest pain or sudden severe pain in the chest which

may increase with deep breathing

- severe light headedness or dizziness
- rapid or irregular heartbeat
- sudden pain, swelling and slight blue discoloration of an extremity
- sudden numbness or weakness of the face, arm or leg, especially on one side of the body
- sudden trouble walking, dizziness, loss of balance or coordination
- sudden confusion, slurred speech or aphasia; sudden partial or complete loss of vision, double vision, painless blurring of vision which can progress to loss of vision
- sudden, severe or prolonged headache with no known cause
- loss of consciousness or fainting with or without seizure
- pain, discomfort, pressure, heaviness, sensation of squeezing or fullness in the chest arm, or below the breastbone
- discomfort radiating to the back, jaw, throat, arm, stomach
- feeling of being full, having indigestion or choking
- sweating, nausea, vomiting extreme weakness and anxiety.

**If you are going to have surgery, tell the surgeon or anaesthetist beforehand that you are taking Levlen ED.** The risk of having blood clots is temporarily increased as a result of major surgery, any surgery to the legs or pelvis, neurosurgery or major trauma. In women who take Levlen ED, the risk may be higher.

In women at risk of prolonged immobilisation (including major surgery, any surgery to the legs or pelvis, neurosurgery, or major trauma), your doctor may tell you to stop taking (in the case of elective surgery at least four weeks in advance) and not resume until two weeks after complete remobilisation. Another method of contraception should be used to avoid unintentional pregnancy. Your doctor may prescribe other treatment (e.g. treatment for blood clots) if Levlen ED has not been discontinued in advance.

**Other risk factors for blood clotting include temporary immobilisation including air travel of greater than 4 hours, particularly in women with other risk factors. Consult your doctor if you plan to air travel for greater than 4 hours. Consult your doctor if you develop high blood pressure while taking Levlen ED – you may be told to stop taking it.**

**If you become pregnant while taking this medicine, tell your doctor immediately.**

**If you vomit within 3 to 4 hours, or have severe diarrhoea after taking a beige active tablet, the active ingredients may not have been completely absorbed. This is like missing a tablet. Follow the advice for missed tablets.**

**If you have unexpected bleeding and it continues, becomes heavy, or occurs again, tell your doctor.** When taking this Pill for the first few months, you can have irregular vaginal bleeding (spotting or breakthrough bleeding) between your periods. You may need to use sanitary products, but continue to take your tablets as normal. Irregular vaginal bleeding usually stops once your body has adjusted to the Pill, usually after about 3 months.

**If you have missed a period, but you have taken all your tablets, it is unlikely that you are pregnant, as long as:**

- you have taken the beige active tablets at the right time
- you have not been taking a medicine(s) that may interfere with your Pill
- you have not vomited or had severe diarrhoea during this cycle.

If this is so, continue to take Levlen ED as usual. If you have any concerns consult your doctor or pharmacist.

**If you miss your period twice in a row, you may be pregnant, even if you have taken the Pill correctly. Stop taking Levlen ED and seek advice from your doctor. You must use a non-hormonal method of contraception, (such as condoms or a diaphragm) until your doctor rules out pregnancy.**

Levlen ED will not protect you from HIV-AIDS or any other sexually transmitted infections (STIs), such as chlamydia, genital herpes, genital warts, gonorrhoea, hepatitis B, human papillomavirus and syphilis.

**To protect yourself from STIs, you will need to use additional barrier contraceptives (e.g. condoms).**

### *Things you must not do*

**Do not take Levlen ED to treat any other conditions, unless your doctor tells you to.**

**Do not give your medicine to anyone else.**

**Do not stop taking your medicine or change the dosage without checking with your doctor.** You may become pregnant

if you are not using any other contraceptive and you stop taking Levlen ED, or do not take a tablet every day.

## SIDE EFFECTS

**Tell your doctor or pharmacist as soon as possible if you do not feel well while you are taking Levlen ED.**

This Pill helps most women, but it may have unwanted side effects in some women.

All medicines can have side effects. Sometimes they are serious, most of the time they are not. You may need medical attention if you get some of the side effects.

**Do not be alarmed by the following lists of side effects. You may not experience any of them.**

**Ask your doctor or pharmacist to answer any questions you may have.**

The following list includes the more common side effects of your Pill. These are usually mild and lessen with time.

**If you notice any of the following side effects and they worry you, tell your doctor or pharmacist:**

- acne
- nausea
- stomach pain
- changes in weight
- headache, including migraines
- mood changes, including depression
- breast tenderness or pain
- hair loss or hair growth.

The following list includes very serious but rare side effects. You may need urgent medical attention or hospitalisation.

**If you experience any of the following, tell your doctor immediately, or go to the Accident and Emergency Department at your nearest hospital:**

- pain in the chest, arm or below the breastbone
- pain or discomfort that goes to your back
- breathlessness and/or difficulty breathing
- swelling, pain or tenderness of one leg
- sudden weakness, numbness or bad 'pins and needles' of the face, arm or leg, especially on one side of the body
- sudden trouble walking, dizziness, loss of balance or coordination
- severe, sudden stomach pains
- a fainting attack or you collapse
- unusual headaches or migraines that are worse than usual
- sudden problems with speaking, seeing or understanding what people are saying to you

The side effects listed above are possible signs of a blood clot (thrombosis).

- jaundice (yellowing skin or yellowing eyes)
- you cough up blood
- breast lumps
- unexplained vaginal bleeding.

**Tell your doctor or pharmacist if you notice anything else that is making you feel unwell.** Other side effects not listed above may also occur in some people.

### *Blood clots and the Pill*

Blood clots may block blood vessels in your body. This type of blood clot is also called thrombosis.

Blood clots sometimes occur in the deep veins of the legs. If a blood clot breaks away from the veins where it has formed, it may reach and block the blood vessels of the lungs, causing pulmonary embolism.

Blood clots can also occur in the blood vessels of the heart (causing a heart attack) or the brain (causing a stroke).

Blood clots are a rare occurrence and can develop whether or not you are taking the Pill. They can also happen during pregnancy. The risk of having blood clots is higher in Pill users than in non-users, but not as high as during pregnancy.

The risk of a blood clot is highest during the first year of taking the Pill for the first time, or after having a break from the Pill for 4 weeks or more.

**If you notice possible signs of a blood clot, stop taking Levlen ED and consult your doctor immediately.** To prevent pregnancy, you must also use additional barrier contraceptive precautions (e.g. condoms or a diaphragm).

**If you are concerned about an increased risk of blood clots while on Levlen ED, speak to your doctor.**

### *Cancer and the Pill*

Breast cancer has been diagnosed slightly more often in women who take the Pill than in women of the same age who do not take the Pill.

This slight increase in the numbers of breast cancer diagnoses gradually disappears during the course of the 10 years after women stop taking the Pill.

It is not known whether the difference is caused by the Pill. It may be that these women were examined more often, so that the breast cancer was noticed earlier.

**It is important that you check your breasts regularly and contact your doctor if you feel any lumps.**

In rare cases benign liver tumours and, even more rarely, malignant liver tumours have been reported in users of the Pill. These tumours may lead to internal bleeding.

**Contact your doctor immediately if you have severe pain in your abdomen.**

Cervical cancer has been reported to occur more often in women who have been taking the Pill for a long time. This finding may not be caused by the Pill, but may be related to sexual behaviour and other factors.

## AFTER TAKING LEVLEN ED

### *Storage*

**Keep your tablets in the blister pack until it is time to take them.** If you take the tablets out of the pack they may not keep well.

**Keep your tablets in a cool dry place where the temperature stays below 30°C.**

**Do not store your tablets or any other medicine in the bathroom, near a sink, or on a window-sill. Do not leave medication in the car.** Heat and damp can destroy some medicines.

**Keep Levlen ED where children cannot reach it.**
A locked cupboard at least one-and-a-half metres above the ground is a good place to store medicines.

### *Disposal*

If your doctor tells you to stop taking this medicine or the expiry date has passed, ask your pharmacist what to do with any medicine that is left over.

**Return any unused medicine to your pharmacist.**

## PRODUCT DESCRIPTION

### *What Levlen ED looks like*

Levlen ED active tablets are beige and round.

Levlen ED inactive tablets are white and round.

Levlen ED comes in a box containing 1 or 4 blister packs. Not all pack sizes may be marketed.

Each blister pack contains 21 beige active tablets and 7 white inactive tablets.

### *Ingredients*

**Each Levlen ED beige active tablet contains:**

**Active ingredients:**
- 30 microgram of ethinylestradiol
- 150 microgram of levonorgestrel

**Inactive ingredients:**
- calcium carbonate
- glycerol
- glycol montanate
- iron oxide yellow
- lactose monohydrate
- macrogol 6000
- magnesium stearate
- maize starch
- povidone
- purified talc
- sucrose
- titanium dioxide

**Each white inactive tablet contains:**
- calcium carbonate
- glycol montanate
- lactose monohydrate
- macrogol 6000
- magnesium stearate
- maize starch
- povidone
- purified talc
- sucrose

**Tablets do not contain gluten. Tablets also do not contain tartrazine or any other azo dyes.**

### *Supplier*

Made in Germany for:

Bayer Australia Ltd
ABN 22 000 138 714
875 Pacific Highway
Pymble NSW 2073

### *Australian Registration Number*

Levlen ED - AUST R 40193

### *Date of Preparation*

March 2020

See TGA website (www.ebs.tga.gov.au) for latest Australian Consumer Medicine Information.

**Missed a pill?**
See the end of this leaflet.

® Registered Trademark of the Bayer Group, Germany
© Bayer Australia Ltd
All rights reserved.

## Summary of advice if you missed a beige active tablet more than 12 hours ago.

| | | | | |
|---|---|---|---|---|
| **Before missing your tablet, did you take beige active tablets for the previous 7 days?** | **No** | Did you have sex in the 7 days before missing the tablet? | → | **No**<br>Take the tablet missed AND use extra barrier precaution for 7 days. If there are fewer than 7 beige active tablets left in the pack, finish the beige active tablets and go straight to the beige active tablets of the next pack. This means you skip the white inactive tablets.<br><br>**Yes**<br>See your Doctor or Pharmacist for advice |
| | **Yes** | Does your pack still have 7 beige active tablets in a row to follow? | → | **No**<br>Take the tablet you missed AND complete taking the beige active tablets. Skip the white inactive tablets. Start your next pack with beige active tablets.<br><br>**Yes**<br>Take the tablet you missed AND complete the pack as normal |
