# Supplementary material for: A simple experiment to improve adherence for taking the oral contraceptive pill: An exploratory study of behavioural mechanisms
Source: Br J Health Psychol. 2025 Mar 3;30(2):e12788. doi: 10.1111/bjhp.12788 (PMC11876489; doi:10.1111/bjhp.12788)
Supplement: Supplementary file 2 — Data S2. [file BJHP-30-0-s002.docx]

**Supplementary Material**

A Simple Experiment to Improve Adherence for Taking the Oral Contraceptive Pill: An Exploratory Study of Behavioural Mechanisms

**Instructions Provided to Each Experimental Condition**

**Group 1 - Control**

Thank you for volunteering to be part of this study and for completing the first questionnaire. For the next 6 weeks please ensure you take your oral contraceptive pill as you currently normally would. You are not required to change anything. The researchers will contact you at the end of the 6 weeks to ask you some questions.

**Group 2 – Credible Information Only**

Thank you for volunteering to be part of this study and for completing the first questionnaire. Please read the following oral contraceptive pill information sheet and for the next 6 weeks try and take your oral contraceptive pill according to this information sheet. You are not required to do anything else. The researchers will contact you at the end of the 6 weeks to ask you some questions.

**Group 3 – Cue Implementation Only**

Thank you for volunteering to be part of this study and for completing the first questionnaire. A cue is something that prompts a behaviour. Can you please spend the next few minutes thinking of a cue that you can match to your oral contraceptive pill, to assist you in remembering to take your pill. It can be anything that you do every day. For example, you may choose brushing your teeth before bed, or placing your pill box on your bedside table so you see it every morning when you wake up. We would like you to pair this cue with you taking your oral contraceptive pill. For the next 6 weeks try and take your daily oral contraceptive pill when you see or experience your chosen cue. The researchers will contact you at the end of the 6 weeks to ask you some questions.

**Group 4 – Credible Information + Cue Implementation**

Thank you for volunteering to be part of this study and for completing the first questionnaire. Please read the following oral contraceptive pill information sheet and after, spend the new few minutes thinking of a cue that you can match to your oral contraceptive pill, to assist you in remembering to take your pill. A cue is something that prompts a behaviour. It can be anything that you do every day. For example, you may choose brushing your teeth before bed, or placing your pill box on your bedside table so you see it every morning when you wake up. We would like you to pair this cue with you taking your oral contraceptive pill. For the next 6 weeks try and take your daily oral contraceptive pill when you see or experience your chosen cue. The researchers will contact you at the end of the 6 weeks to ask you some questions.
