# Supplementary material for: A simple experiment to improve adherence for taking the oral contraceptive pill: An exploratory study of behavioural mechanisms
Source: Br J Health Psychol. 2025 Mar 3;30(2):e12788. doi: 10.1111/bjhp.12788 (PMC11876489; doi:10.1111/bjhp.12788)
Supplement: Supplementary file 3 — Data S3. [file BJHP-30-0-s003.pdf]

MICROLUT® (MY·crow·loot)

Contraceptive tablets for women

levonorgestrel

Consumer Medicine Information

WHAT IS IN THIS LEAFLET

This leaflet answers some common questions about Microlut. It does not contain all the available information. It does not take the place of talking to your doctor or pharmacist.

All medicines have risks and benefits. Your doctor has weighed the risks of you taking Microlut against the benefits they expect it will have for you.

If you have any concerns, or are unsure about taking this medicine, ask your doctor or pharmacist for more advice.

Keep this leaflet with the medicine.
You may need to read it again.

WHAT MICROLUT IS USED FOR

Microlut is an oral progestogen-only contraceptive, commonly known as the 'Mini-pill'.

Microlut is used to prevent pregnancy.

When taken correctly, it prevents you from becoming pregnant in several ways, including:

- changing the cervical mucus consistency, making it more difficult for the sperm to reach the egg
- changing the lining of the uterus, making it less suitable for implantation
- impairing mid-cycle functions, which may contribute to contraceptive action.

Like all oral contraceptives (OC), Microlut is intended to prevent pregnancy. It does not protect against HIV infection (AIDS) and other sexually transmitted infections.

Ask your doctor if you have any questions about why this medicine has been prescribed for you.
Your doctor may have prescribed it for another reason.

BEFORE YOU TAKE MICROLUT

When you must not take it

Do not take Microlut if you have an allergy to:

- levonorgestrel the active ingredient in Microlut
- any of the ingredients listed at the end of this leaflet.

Some of the symptoms of an allergic reaction may include:

- shortness of breath
- wheezing or difficulty in breathing
- swelling of the face, lips, tongue or other parts of the body
- rash, itching or hives on the skin.

Do not take Microlut if you have or have had a blood clot in:

- the blood vessels of the legs (deep vein thrombosis - DVT)
- the lungs (pulmonary embolism - PE)
- the heart (heart attack)
- the brain (stroke)
- other parts of the body.

Do not take Microlut if you are concerned about an increased risk of blood clots. Blood clots are rare. Very occasionally blood clots may cause serious permanent disability, and may even be fatal.

You are more at risk of having a blood clot when you take the Mini-pill. However, the risk of having a blood clot when taking the Mini-pill is less than the risk of having a blood clot during pregnancy.

Do not take Microlut if you are concerned about an increased risk of blood clots because of age or smoking.
The risk of having a heart attack or stroke increases as you get older. It also increases if you smoke. You should stop smoking when taking the Mini-pill, especially if you are older than 35 years of age.

Do not take Microlut if you have, or have had:

- diabetes mellitus with blood vessel damage
- severe liver disease and your liver function has not returned to normal
- cancer that may grow under the influence of sex hormones (e.g. of the breast or the genital organs)
- benign or malignant liver tumour
- unexplained vaginal bleeding.

Do not take this medicine if you are pregnant or think you might be pregnant.

Do not give this medicine to a child.
Microlut is not intended for use in females whose periods have not yet started.

Do not take this medicine after the expiry date printed on the pack and blister.
The expiry date is printed on the carton and on each blister after "EXP" (e.g. 11 18 refers to November 2018). The expiry date refers to the last day of that month. If it has expired return it to your pharmacist for disposal.

Do not take this medicine if the packaging is torn or shows signs of tampering.
If the packaging is damaged, return it to your pharmacist for disposal.

If you are not sure whether you should start taking this medicine, talk to your doctor.

Before you start to take it

Tell your doctor if you have allergies to any other medicines, foods, preservatives or dyes.

Tell your doctor if:

- you smoke
- you have abdominal pain with infrequent and/ or irregular periods
- you have ever had an extra uterine/ectopic pregnancy (where an embryo has developed outside the womb) or an impairment in your fallopian tube function (e.g. caused by inflammation).
- you or anyone in your immediate family has had blood clots in the legs (DVT) or lungs (PE), a heart attack, a stroke, breast cancer or high cholesterol.

Tell your doctor if you have, or have had any of the following medical conditions:

- diabetes
- high blood pressure
- kidney or heart problems
- migraine
- asthma
- epilepsy
- depression

Ask your doctor to check if you:

- are overweight
- have high cholesterol or triglycerides
- have liver disease
- have gall bladder disease
- have a condition that occurred for the first time, or worsened during pregnancy or previous use of sex hormones (e.g. cholestatic jaundice and/or pruritis (itching)
- have chloasma (yellowish-brown pigmentation patches on the skin, particularly of the face) – if so, avoid exposure to the sun or ultraviolet radiation.

If any of the above conditions appear for the first time, recur or worsen while taking Microlut, you should contact your doctor.

Microlut contains lactose. If you have an intolerance to some sugars, contact your doctor before you start taking Microlut.

If you have not told your doctor about any of the above, tell him/her before you start taking Microlut.

Taking other medicines

Tell your doctor or pharmacist if you are taking any other medicines, including any that you get without a prescription from your pharmacy, supermarket or health food shop.
Some medicines and Microlut may interfere with each other. These include:

- medicines used to treat tuberculosis such as rifampicin, rifabutin
- a class of antibiotics known as macrolides, such as clarithromycin, erythromycin
- medicines used to treat fungal infections, such as ketoconazole, griseofulvin
- medicines used to treat HIV, such as ritonavir, nevirapine
- some medicines used to treat Hepatitis C Virus (HCV), such as boceprevir, telaprevir
- medicines used to treat epilepsy such as phenytoin, primidone, barbiturates (e.g. phenobarbitone), carbamazepine, oxcarbazepine, topiramate, felbamate, lamotrigine
- antibiotics (e.g. penicillins, nitrofurantoin, tetracycline)
- cyclosporin, an immunosuppressant medicine
- some medicines used to treat high blood pressure, chest pain or irregular

heartbeats such as diltiazem, verapamil
- herbal medicines containing St John's Wort
- grapefruit juice.

These medicines may be affected by Microlut, or may affect how well it works. Your doctor may need to alter the dose of your medicine, or prescribe a different medicine.

You may need to use additional barrier methods of contraception (such as condoms or a diaphragm) while you are taking any of these medicines and for some time after stopping them. Your doctor will be able to advise you on how long you will need to use additional contraceptive methods.
Your doctor and pharmacist have more information on medicines that you need to be careful with or avoid while taking this medicine.

HOW TO TAKE MICROLUT

Follow all directions given to you by your doctor or pharmacist carefully.
They may differ from the information contained in this leaflet.

If you do not understand the instructions on the pack, on the pharmacist label or in this leaflet, ask your doctor or pharmacist for help.

How to take it

Take one tablet daily at the same time every day. You must take Microlut every day regardless of how often you have sex. This will also help you remember when to take it.

An interval of exactly 24 hours should be maintained between tablets. This interval must not be exceeded by more than 3 hours.

Even if one tablet is taken late (i.e. more than 3 hours later than when it should have been taken) or if one tablet is missed altogether, protection against pregnancy may be impaired.

Swallow the tablet whole with water. It does not matter if you take it before or after food.

Each blister pack is marked with the day of the week. **Take your first tablet from the blister pack corresponding to the day of the week.**

Follow the direction of the arrows on the blister pack until all the tablets have been taken. Each blister pack is marked with the day of the week.

Tablets must be taken for 28 consecutive days. There is no break between packs. This means that when the first pack is finished the next should be started without interruption.

If you do not understand the instructions on the blister pack, ask your doctor or pharmacist for help.

Always start a new blister pack on the same day of the week as your previous pack.

Taking Microlut for the first time

If you are starting Microlut after a natural cycle, and you have not used a hormonal contraceptive in the past month, start on the first day of your period, i.e. on the first day of your menstrual bleeding.

Your doctor will advise you when to start if you:
- are taking Microlut after having a baby
- are breast-feeding
- have had a miscarriage or an abortion.

Changing from another contraceptive

Changing from a combined oral contraceptive:
Start taking Microlut on the day after taking the last active tablet in your previous pill pack. Do not take the inactive (sugar) tablets of your previous pack.

If you are not sure which were active/inactive tablets in your previous pill pack, ask your doctor or pharmacist.
Your previous pill pack may have different colour tablets to those of Microlut.

Changing from a progestogen-only pill ('minipill'):
Stop taking the previous Mini-pill on any day and start taking Microlut at the same time the next day, with out any break between Mini-pills.

Changing from a progesterone only injection or implant:
Start taking Microlut when your next injection is due, or on the day that your implant is removed.

You must also use additional barrier contraceptive precautions (e.g. condoms or a diaphragm) for the first 7 days of tablet-taking when having intercourse.

Stopping Microlut

You can stop taking Microlut at any time. If you are considering becoming pregnant, it is recommended that you begin taking a vitamin supplement containing folic acid. It is best that you start taking folic acid tablets before you stop taking Microlut and not stop until your doctor advises this. Seek advice from your doctor or pharmacist about suitable supplements. It is both safe and recommended that you take folic acid during pregnancy.

Additional contraceptive precautions

When additional contraceptive precautions are required you should either abstain from sex, or use a barrier method of contraception, a cap (or diaphragm) plus spermicide, or a condom. Rhythm methods are not advised as Microlut disrupts the cyclical changes associated with the natural menstrual cycle e.g. changes in temperature and cervical mucus.

If you forget to take it

If you miss a tablet, take the missed tablet as soon as you remember, even if this means taking two tablets at the same time. Then continue to take your tablets at the usual time. In addition, you should also use additional barrier contraceptive precautions (e.g. condoms or a diaphragm) for the next 7 days.

If you have had sexual intercourse in the week before, taking your tablet(s) late or missed a tablet(s), there is a high possibility of becoming pregnant. **Seek advice from your doctor or pharmacist about what to do.**

Please refer to the table at the end of this leaflet "Summary of advice when late taking or missing a Mini-pill".

Ask your doctor or pharmacist to answer any questions you may have.

If you take too much (overdose)

Immediately telephone your doctor or the Poisons Information Centre (Australia: 13 11 26) for advice, or go to the Accident and Emergency Department at your nearest hospital, if you think that you or anyone else may have taken too much Microlut. Do this even if there are no signs of discomfort or poisoning. You may need urgent medical attention.

WHILE YOU ARE TAKING MICROLUT

Things you must do

Tell any doctors, dentists and pharmacists who treat you that you are taking this medicine.

If you are about to have any blood tests, tell your doctor that you are taking this medicine. It may interfere with the results of some tests.

Have regular check-ups with your doctor.
When you are taking Microlut, your doctor will tell you to return for regular check ups, including getting a Pap smear test. Your doctor will advise how often you need a Pap smear test. A Pap smear test can detect abnormal cells lining the cervix. Sometimes abnormal cells can progress to cancer.

If you are about to start on any new medicine, remind your doctor and pharmacist that you are taking Microlut.

Stop taking Microlut and see your doctor immediately if you notice possible signs of thrombosis. These include:

- an unusual cough
- severe pain or heaviness in the chest
- breathlessness
- any unusual, severe, or prolonged headache or migraine attack
- partial or complete loss of vision, or double vision
- slurring or speech disability
- sudden changes to your hearing, sense of smell, or taste
- dizziness or fainting
- weakness or numbness in any part of your body
- severe pain in your abdomen
- severe pain, swelling or discolouration in either of your legs.

If you are going to have surgery, tell the surgeon or anaesthetist beforehand that you are taking Microlut. The risk of having DVT is temporarily increased as a result of an operation or immobilisation (for example, when you have your leg(s) in plaster/splints). In women who take the Mini-pill, the risk may be higher.

Your doctor may tell you to stop taking the Mini-pill several weeks before surgery, or at the time of immobilisation, and when you can start taking the Mini-pill again. If you notice possible signs of a thrombosis, stop taking the Mini-pill and consult your doctor immediately.

Consult your doctor if you develop high blood pressure while taking Microlut – you may be told to stop taking it.

If you become pregnant while taking this medicine, tell your doctor immediately.

If you vomit within 3-4 hours or have severe diarrhoea after taking a tablet, the active ingredients may not have been completely absorbed. This is like missing a tablet. Follow the advice for missed tablets.

If you have unexpected bleeding and it continues, becomes heavy, or occurs again, tell your doctor.
When taking these tablets for the first few months, you can have irregular vaginal bleeding (spotting or breakthrough bleeding) between your periods. You may need to use sanitary protection, but continue to take your tablets as normal. Irregular vaginal bleeding usually stops once your body has adjusted to the Mini-pill, usually after about 3 months.

If you have missed a period, but you have taken all your tablets, it is unlikely that you are pregnant as long as:

- you have taken the tablets at the right time
- you have not been taking a medicine(s) that may interfere with your Mini-pill
- you have not vomited or had severe diarrhoea during this cycle.

If this is so, continue to take Microlut as usual. If you have any concerns consult your doctor or pharmacist.

If you miss your period twice in a row, you may be pregnant even if you have taken Microlut correctly. Stop taking Microlut and seek advice from your doctor. You must use a non-hormonal method of contraception (such as condoms, or a diaphragm) until your doctor rules out pregnancy.

Microlut will not protect you from HIV-AIDS or any other sexually transmitted infection (STIs), such as chlamydia, genital herpes, genital warts, gonorrhoea, hepatitis B, human papillomavirus and syphilis.

To protect yourself from STIs, you will need to use additional barrier contraceptives (e.g. condoms).

Things you must not do

Do not take Microlut to treat any other conditions, unless your doctor tells you to.

Do not give your medicine to anyone else.

Do not stop taking your medicine or change the dosage without checking with your doctor.
You may become pregnant if you are not using any other contraceptive and you stop taking Microlut, or do not take a tablet every day.

SIDE EFFECTS

Tell your doctor or pharmacist as soon as possible if you do not feel well while you are taking Microlut.

This medicine helps most women, but it may have unwanted side effects in some women.

All medicines can have side effects. Sometimes they are serious, most of the time they are not. You may need medical attention if you get some of the side effects.

Do not be alarmed by the following lists of side effects. You may not experience any of them.

Ask your doctor or pharmacist to answer any questions you may have. Tell your doctor or pharmacist if you feel unwell.
Other side effects not listed on the following pages may also occur in some people.

The following list includes the more common side effects of your medicine. These are usually mild and lessen with time.

If you notice any of the following side effects and they worry you, tell your doctor or pharmacist:

- nausea, vomiting
- stomach pain together with bleeding irregularities including more frequent, less frequent or no bleeding
- headache, including migraines
- dizziness
- mood changes, including depression
- breast tenderness or pain.

The following list includes very serious but rare side effects. You may need urgent medical attention or hospitalisation.

If you experience any of the following, tell your doctor immediately, or go to the Emergency Department at your nearest hospital:

- pain in the chest, arm or below the breastbone

- discomfort radiating to the back
- breathlessness and/or difficulty breathing
- swelling, pain or tenderness in one leg
- sudden weakness, numbness or bad 'pins and needles' of the face, arm or leg, especially on one side of the body
- sudden trouble walking, dizziness, loss of balance or coordination
- severe, sudden stomach pains
- a fainting attack, or you collapse
- unusual headaches or migraines that are worse than usual
- sudden problems with speech, understanding or eyesight

The side effects listed above are possible signs of a blood clot (thrombosis).

- jaundice (yellowing skin or yellowing eyes)
- you cough up blood
- breast lumps
- unexplained lower abdominal pain, including loss of periods or heavy bleeding. In rare cases pregnancies may occur during use of the mini-pill. These pregnancies are more likely to be extrauterine/ectopic (where the embryo grows outside of the womb)
- unexplained vaginal bleeding.

Tell your doctor or pharmacist if you notice anything else that is making you feel unwell. Other side effects not listed above may also occur in some people.

Thrombosis and the Mini-pill

Thrombosis is the formation of a blood clot that may block a blood vessel.

Thrombosis sometimes occurs in the deep veins of the legs (DVT). If a blood clot breaks away from the veins where it has formed, it may reach and block the arteries of the lungs, causing pulmonary embolism (PE).

Blood clots can also occur in the blood vessels of the heart (causing a heart attack) or the brain (causing a stroke).

Blood clots are a rare occurrence and can develop whether or not you are taking the Mini-pill. They can also happen during pregnancy. The risk of having blood clots is higher in Mini-pill users than in non-users, but not as high as during pregnancy.

The risk of a blood clot is highest during the first year of taking the Mini-pill for the first time, or after having a break from the Mini-pill for 4 weeks or more.

If you notice possible signs of a blood clot, stop taking Microlut and consult your doctor immediately.

If you are concerned about an increased risk of blood clots while on Microlut, speak to your doctor.

Cancer and the Mini-pill

Breast cancer has been diagnosed slightly more often in women who use oral contraception than in women of the same age who do not. This slight increase in the numbers of breast cancer diagnoses gradually disappears during the course of the 10 years after women stop taking the Mini-pill.

It is not known whether the difference is caused by the Mini-pill. It may be that these women were examined more often, so that the breast cancer was noticed earlier.
It is important that you check your breasts regularly and contact your doctor if you feel any lump.

In rare cases benign liver tumours and, even more rarely, malignant liver tumours have been reported in users of the Mini-pill. These tumours may lead to internal bleeding.

Contact your doctor immediately if you have severe pain in your abdomen.

Cervical cancer has been reported to occur more often in women who have been taking the Mini-pill for a long time. This finding may not be caused by the Mini-pill, but may be related to sexual behaviour and other factors.

AFTER TAKING MICROLUT

Storage

Keep your tablets in the blister pack until it is time to take them.
If you take the tablets out of the pack they may not keep well.

Keep your tablets in a cool dry place where the temperature stays below 30°C.

Do not store your tablets or any other medicine in the bathroom, near a sink, or on a window-sill.

Do not leave medication in the car.
Heat and damp can destroy some medicines.

Keep Microlut where children cannot reach it.
A locked cupboard at least one-and-a-half metres above

the ground is a good place to store medicines.

Disposal

If your doctor tells you to stop taking this medicine or the expiry date has passed, ask your pharmacist what to do with any medicine that is left over.

Return any unused medicine to your pharmacist.

PRODUCT DESCRIPTION

What it looks like

Microlut comes in a box containing 1 or 4 blister packs. Each blister pack contains 28 white active tablets. Not all pack sizes may be marketed.

Ingredients

Active ingredients:

- Microlut – 30 microgram of levonorgestrel per tablet

Each tablet also contains:

- calcium carbonate
- glycol montanate
- lactose monohydrate
- macrogol 6000
- magnesium stearate
- maize starch
- povidone
- purified talc
- sucrose

Tablets do not contain gluten. Tablets also do not contain tartrazine or any other azo dyes.

Supplier

Made in Germany for:

Bayer Australia Ltd
ABN 22 000 138 714
875 Pacific Highway
Pymble NSW 2073

Australian Registration Number

Microlut - AUST R 10696

Date of Preparation

February 2017

See TGA website (www.ebs.tga.gov.au) for latest Australian Consumer Medicine Information.

® Registered Trademark of the Bayer Group, Germany
© Bayer Australia Ltd
All rights reserved.

Summary of advice when late taking *or* missing a Mini-pill

**If it has been more than 27 hours since your last tablet was taken <u>or</u> you have missed a tablet, then:**	• Take the tablet you missed even if this means taking two at the same time. Use barrier contraception for the next 7 days.<br>• Continue taking tablets at usual time.	• If you had sex in the 7 days prior to taking your late tablet or missed tablet, the risk of pregnancy is increased.<br>• Contact your doctor or pharmacist for advice
